# Coronary microvascular dysfunction: a key step in the development of uraemic cardiomyopathy?

**DOI:** 10.1136/heartjnl-2019-315138

**Published:** 2019-06-25

**Authors:** Ashwin Radhakrishnan, Luke C Pickup, Anna M Price, Jonathan P Law, Nicola C Edwards, Richard P Steeds, Charles J Ferro, Jonathan N Townend

**Affiliations:** 1 Birmingham Cardio-Renal Group, Institute of Cardiovascular Sciences, University of Birmingham, Birmingham, UK; 2 Department of Cardiology, Queen Elizabeth Hospital Birmingham, Birmingham, UK; 3 Department of Nephrology, Queen Elizabeth Hospital Birmingham, Birmingham, UK; 4 Green Lane Cardiovascular Service, Auckland City Hospital, Auckland, New Zealand

**Keywords:** myocardial disease

## Abstract

The syndrome of uraemic cardiomyopathy, characterised by left ventricular hypertrophy, diffuse fibrosis and systolic and diastolic dysfunction, is common in chronic kidney disease and is associated with an increased risk of cardiovascular morbidity and mortality. The pathophysiological mechanisms leading to uraemic cardiomyopathy are not fully understood. We suggest that coronary microvascular dysfunction may be a key mediator in the development of uraemic cardiomyopathy, a phenomenon that is prevalent in other myocardial diseases that share phenotypical similarities with uraemic cardiomyopathy such as hypertrophic cardiomyopathy and heart failure with preserved ejection fraction. Here, we review the current understanding of uraemic cardiomyopathy, highlight different methods of assessing coronary microvascular function and evaluate the current evidence for coronary microvascular dysfunction in chronic kidney disease.

## Introduction

Chronic kidney disease (CKD) is common, affecting one in seven of Western populations.w1 Usually, it is mild and there is little risk of progression to end-stage renal disease (ESRD), but the risk of adverse cardiovascular events is elevated. There is a well-documented graded inverse relationship between cardiovascular risk and estimated glomerular filtration rate (eGFR) that is independent of age, sex and other risk factors.^1^ Patients with CKD have an increased risk of coronary artery disease and an even higher risk of death from heart failure, arrhythmias and sudden death, which rises steeply with more severe CKD.^2^ In ESRD, the individual cardiovascular risk is extreme but the public health burden lies in early-stage CKD because of its much higher prevalence.

10.1136/heartjnl-2019-315138.supp1 Supplementary data

Pathological structural and functional remodelling occurs in the heart and vascular system in CKD. Left ventricular hypertrophy (LVH) is found in over 70% of patients with ESRD and other manifestations of heart muscle disease such as focal scarring and diffuse interstitial fibrosis (DIF) frequently occur, comprising the phenotype of uraemic cardiomyopathy.^3^ These findings are also present to a lesser degree in early-stage disease.^4^ Hypertension is near universal. Vascular calcification is common and results from accelerated atherosclerosis (intimal disease) and arteriosclerosis (medial disease).^5^ Regardless of the vascular bed affected, these changes confer elevated cardiac risk by increasing arterial stiffness, which can be measured by pulse wave velocity and augmentation index.^5^ These arterial changes increase LV afterload which, together with humoral hypertrophic and profibrotic stimuli, lead to the syndrome of uraemic cardiomyopathy.^5^ As eGFR declines, the severity of this myocardial disease increases, possibly explaining the very high risk of death due to heart failure and sudden (presumed arrhythmic) cardiac death in ESRD.

## Uraemic cardiomyopathy

The syndrome of uraemic cardiomyopathy, characterised by LVH, DIF, focal scarring and systolic and diastolic dysfunction, is highly prevalent in ESRD.^2 3^ Uraemic cardiomyopathy has been well described in recent years, mainly using cardiac MRI (CMR).^3 6 7^ The increased LV mass seen in ESRD is due to both myocyte hypertrophy and an expansion of the interstitial space caused by DIF. Myocardial biopsy studies show that many subjects with ESRD have myocardial appearances resembling the dilated phase of hypertrophic cardiomyopathy (HCM) with severe myocyte hypertrophy, myocyte disarray and extensive DIF.^8^ This fibrotic process can be demonstrated non-invasively on CMR by T1 mapping; a technique that quantifies the relaxation time of protons on inversion recovery prepared images (T1 times) by using analytical expression of image-based signal intensities.w2 T1 relaxation times increase with interstitial expansion due to oedema, infarction, infiltration and fibrosis, and thus provide a sensitive, though non-specific marker of different myocardial disease states.w2 Interstitial fibrosis, identified by elevated T1 times, correlates with histological specimens in hypertrophic and dilated cardiomyopathy and valvular heart disease.w3 Patients with ESRD also have increased T1 times, in keeping with these other myocardial disease states.^6 7^ The fibrotic process occurs early in CKD, with elevated T1 times documented in patients with stages 2–3 CKD compared with age-matched and sex-matched controls.^4^ DIF is probably responsible for reduced systolic function, reflected by reduced markers of deformation,w4 but causes severe diastolic dysfunction as tissue collagen deposition affects viscoelasticity of the myocardium leading to impaired relaxation, diastolic recoil and passive stiffness.w5 It is believed to be a major cause of the clinical syndrome of heart failure and of the increased risk of arrhythmogenesis seen in uraemic cardiomyopathy.^2^

## Mediators of adverse cardiac remodelling in CKD

The development of uraemic cardiomyopathy is likely to be multifactorial. Haemodynamic factors include increased afterload due to hypertension and arterial stiffness, and increased preload due to anaemia and sodium overload.^2^ A wide range of humoral and local factors are involved. Activation of the renin–angiotensin–aldosterone system, hyperuricaemia, uraemic toxins such as asymmetric dimethylarginine, hyperphosphataemia, abnormal bone mineral metabolism, elevated levels of hormones that regulate phosphate (parathyroid hormone and fibroblast growth factor-23 (FGF-23)), oxidative stress and chronic low-grade inflammation have all been implicated in the development of myocardial hypertrophy, fibrosis and increased cardiovascular mortality.^2^

A common consequence of these disparate mediators may be the development of pathological changes in the coronary microcirculation, a phenomenon that is evident in other myocardial disease states,w6 and requires further investigation.

## The coronary microcirculation and myocardial disease

**Figure 1:**
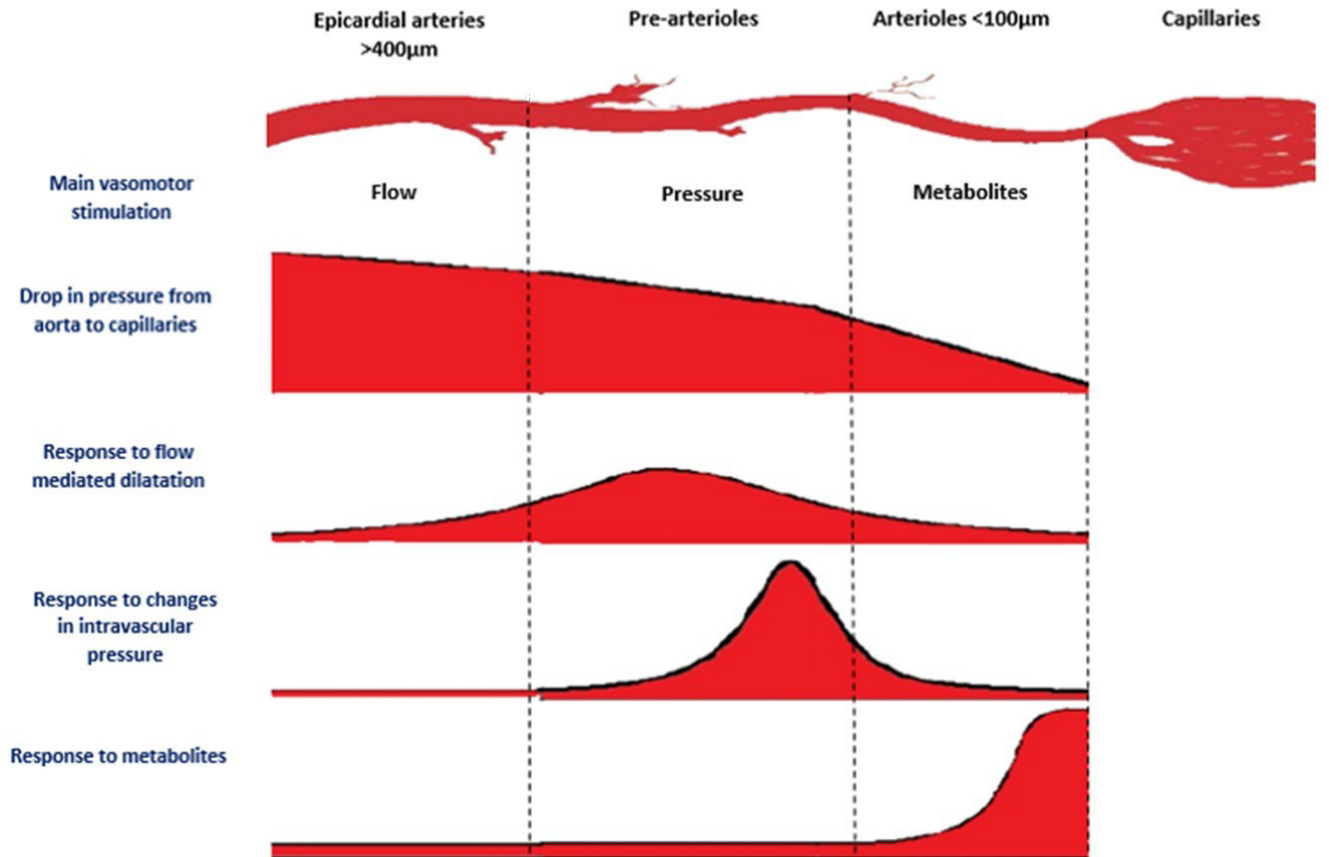
Functional anatomy of the coronary circulation. Adapted from De Bruyne *et al.w6*

Chilian proposed an elegant model of the coronary circulation consisting of three anatomically distinct but functionally interlinked compartments (figure 1).w7

The proximal compartment consists of large epicardial coronary arteries that function as capacitance vessels and respond to shear forces by endothelial mediated dilatation. The middle compartment consists of pre-arterioles that are characterised by a measurable pressure drop along their length. The distal compartment consists of the intramural arterioles that have diameters <100 µm, have high resting tone and are responsible for the majority of coronary vascular resistance.^9^ They dilate in response to changes in myocardial oxygen consumption. Vasoactive mediators such as adenosine and hydrogen peroxide act directly on these vessels to produce vasodilatation.w8 Endothelium-dependent mechanisms involving nitric oxide and endothelium derived relaxing factors are also important, with animal studies showing attenuated vasodilatation of the coronary microvasculature when nitric oxide synthesis is inhibited.w8 Finally, the capillary bed delivers oxygen and substrates to the myocytes. Thus, the coronary circulation matches myocardial oxygen demand with supply via a complex interplay between myogenic tone, metabolic signals, circulating hormones and the intrinsic properties of the endothelium.w7

Abnormalities of all of these coronary vessels are seen in uraemia with atherosclerosis and medial thickening and calcification of the epicardial vessels, and medial hypertrophy and a reduction in the cross-sectional surface area of the pre-arterioles.^5^ Myocyte–capillary mismatch and reduced LV capillary density have also been demonstrated in uraemic hearts in both animal models and postmortem human studies.w9 w10

Abnormalities of coronary microvascular function are evident in myocardial disease states such as HCM and heart failure with preserved ejection fraction (HFpEF) that, like uraemic cardiomyopathy, are characterised by hypertrophy and fibrosis. In HCM, studies using positron emission tomography (PET) have documented impaired microvascular function.w11 This predicts clinical consequences including reduced LV systolic function, adverse ventricular remodelling, ventricular arrhythmias, clinical heart failure and cardiovascular death.^9^ w11 Similarly in HFpEF, coronary microvascular dysfunction (CMD) is common with a recent multicentre study identifying CMD in 75% of patients. This was associated with kidney damage, as measured by albuminuria, as well as a higher N-terminal pro-brain natriuretic peptide and systemic arterial dysfunction.w6

**Figure 2:**
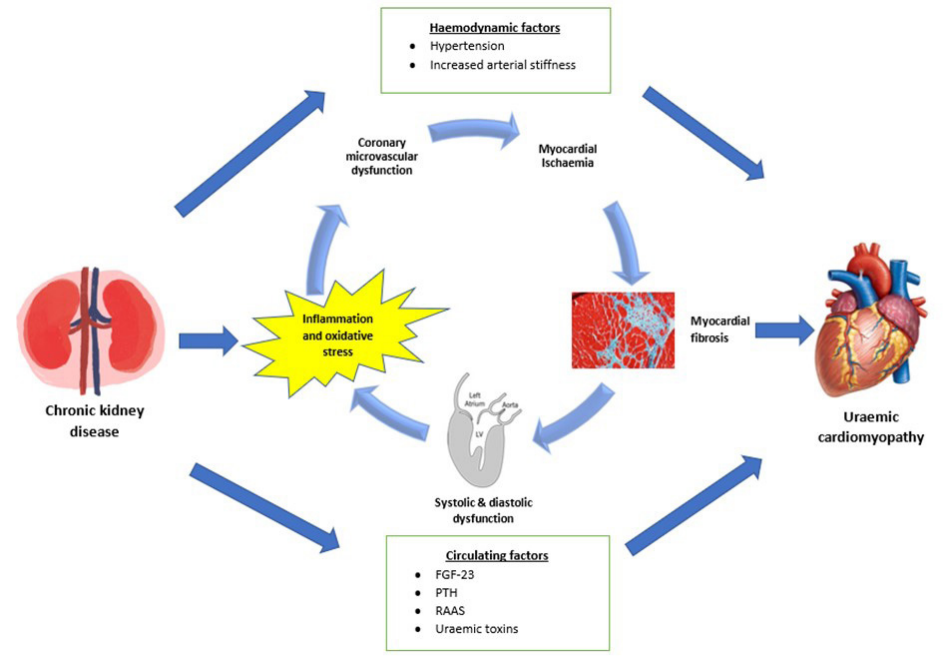
Proposed mechanism of uraemic cardiomyopathy. FGF-23, fibroblast growth factor-23; PTH, parathyroid hormone; RAAS, renin-angiotensin-aldosterone system.

Although not fully understood, a paradigm is emerging which holds that risk factors such as obesity, hypertension, hyperglycaemia and we suggest kidney dysfunction cause CMD, probably as a result of inflammation and oxidative stress. The consequent failure to match myocardial blood flow (MBF) with demand results in widespread ischaemia, DIF, ventricular remodelling and systolic and diastolic dysfunction.w12 In CKD, the effect is likely to be exacerbated by hypertension, increased arterial stiffness and humoral factors such as FGF-23 and aldosterone leading to the clinical syndrome of uraemic cardiomyopathy.^5^ It is not clear if CMD is the cause or consequence of myocardial disease in uraemic cardiomyopathy. However, it is plausible that the relationship between myocardial fibrosis and CMD is reciprocal and a vicious circle is initiated in which both factors exacerbate each other causing progressive ischaemia and myocardial dysfunction leading to heart failure, arrhythmia and death (figure 2).w12

## Methods of assessing coronary microvascular function

**Figure 3:**
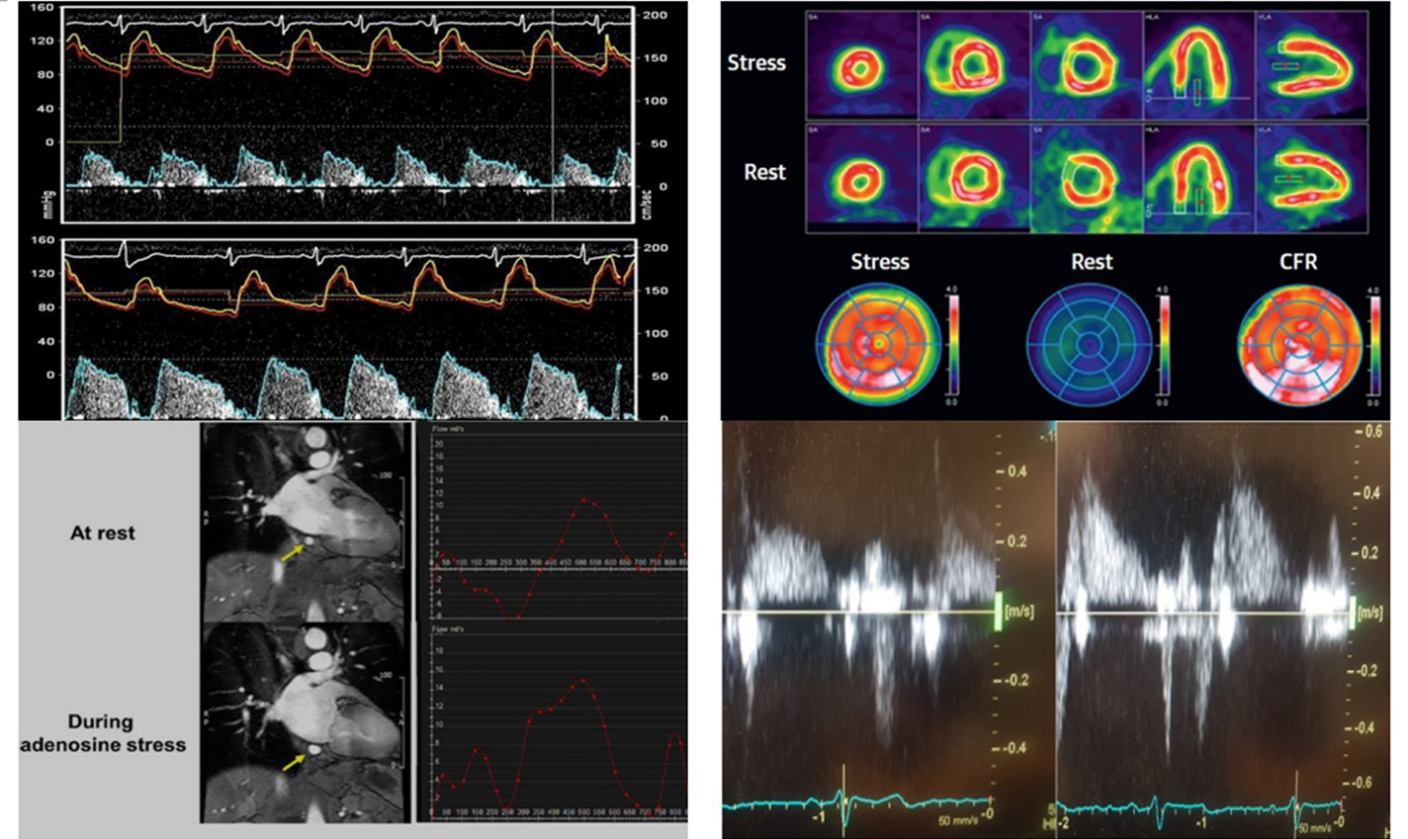
Different methods of assessing CFR: intracoronary Doppler angiography (top right) showing CFR of 1.8 in a patient with coronary artery disease, PET (top left), MRI coronary sinus flow (bottom left) and Doppler transthoracic echocardiogram (bottom right) showing CFR of 2.12 in a patient with chronic kidney disease stage 4. Adapted from Amier *et al*, w16 Feher *et al w17* and Nakamori *et al*.w18 CFR, coronary flow reserve; PET, positron emission tomography.

**Figure 4:**
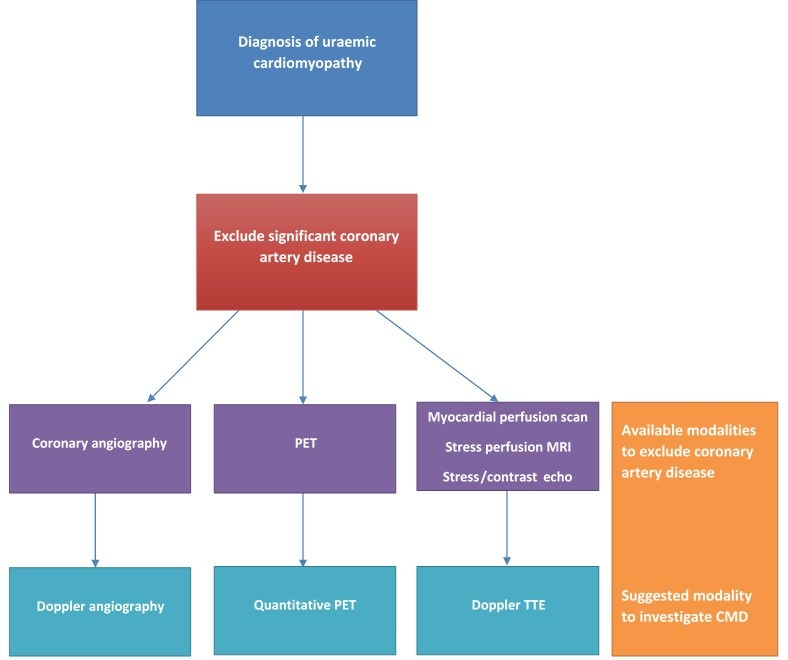
Proposed diagnostic algorithm for coronary microvascular dysfunction (CMD) in uraemic cardiomyopathy. PET, positron emission tomography; TTE, transthoracic echocardiography.

The coronary microcirculation cannot be directly visualised in vivo. All assessments depend on indirect measures of microvascular function. Coronary flow reserve (CFR) is the most widely reported parameter and has been measured using many different techniques (figure 3), which are summarised below and in table 1. To calculate CFR, hyperaemia is induced, usually with a pharmacological vasodilator, and CFR is measured as the ratio of maximal hyperaemic to resting flow. Adenosine is the most commonly used agent, as it is safe with a rapid onset and offset of action.w13 In normal subjects, coronary flow can increase up to fivefold and should at least double with hyperaemic stimuli. Thus, a CFR <2 is considered abnormal.w13 w14 CFR reflects both epicardial coronary artery disease as well as microvascular function. Therefore, exclusion of significant coronary artery disease is required before reduced CFR can be attributed to CMD.^9^ This is often difficult without angiography and is a limitation of many studies. A diagnostic algorithm for CMD in uraemic cardiomyopathy is suggested in figure 4.

**Table 1:** Summary of advantages and disadvantages of different modalities used to assess coronary flow reserve

Modality	Advantages	Disadvantages
Invasive angiography (Doppler and thermodilution)	Definitive exclusion of epicardial coronary artery disease Widely available	Invasive procedure Ionising radiation
Positron emission tomography	Non-invasive Can assess myocardial ischaemia and scar Allows calculation of regional and global myocardial blood flow	Ionising radiation Not widely available in UK
Coronary sinus flow	Non-invasive Sequences and analysis are quick to perform	Contraindications to MRI limit its widespread use
First pass perfusion	Non-invasive Can assess myocardial ischaemia and scar Myocardial viability can be ascertained	Requires gadolinium limiting its utility in chronic kidney disease Scan sequences can be lengthy to perform and analyse Contraindications to MRI limit its widespread use
Stress T1 mapping	Non-invasive Provides additional myocardial tissue characterisation	Contraindications to MRI limit its widespread use Not well validated
Doppler transthoracic echo	Non-invasive Cheap Portable	Only assesses left anterior descending artery territory
Myocardial contrast echo	Non-invasive Cheap Portable Allows calculation of regional and global myocardial blood flow	Requires good acoustic windows

### Invasive coronary angiography

CFR can be assessed during invasive coronary angiography. Two different methods exist but both expose patients to infrequent but significant risks including vascular injury, contrast nephropathy and death.

### Doppler guidewire

An angioplasty wire tipped with a high frequency piezoelectric Doppler transducer can be used to measure flow velocities in a coronary artery at rest and at hyperaemia. CFR is calculated as the ratio of hyperaemic/resting flow.w14

### Intracoronary thermodilution

CFR can be assessed via thermodilution. A pressure wire is positioned in the distal third of a target vessel. The shaft of the pressure wire acts as a proximal thermistor while the sensor at its tip acts as a distal thermistor. Normal saline at room temperature is injected down the coronary artery and its transit time is measured by thermodilution. CFR is the ratio of hyperaemic transit time/baseline transit time. This technique correlates well with Doppler flow derived CFR.w15

### Positron emission tomography

The non-invasive ‘gold-standard’ method of assessing CFR is quantitative PET. Absolute values of MBF at rest and during hyperaemia can be calculated. Advantages of PET include its ability to assess regional blood flow, myocardial scar and myocardial ischaemia as well as CFR.^10^ Disadvantages include exposure to ionising radiation, high cost, difficulties in accessing radio-isotopes and the relative unavailability of the technique.

### MRI

MRI is emerging as a useful tool for the non-invasive assessment of CFR, although it remains less validated than other imaging modalities. Methods include:

### Coronary sinus flow

The majority of blood from epicardial ventricular veins drains into the coronary sinus, which can be visualised on MRI using velocity encoded cine sequences. CFR is the ratio of blood in the coronary sinus after hyperaemia compared with baseline.w16

### First pass perfusion

Myocardial perfusion is recorded in dedicated basal, mid-ventricular and apical short axis slices at rest and during stress. The ratio of the maximal up-slopes of signal intensity during vasodilatation over resting condition is defined as myocardial perfusion reserve.^10^ Perfusion defects can be identified to help localise coronary artery lesions and assessments of viability can be made using late gadolinium enhancement. However, the need for gadolinium limits its utility in CKD.

### Stress T1 mapping

T1 relaxation times of tissues are prolonged by increased water content. Coronary vasodilatation, by increased myocardial blood volume, would be expected to prolong T1 times.w17 Using this principle, measurement of rest and stress T1 times provide an indirect indication of increased MBF and myocardial perfusion reserve.w17

### Doppler transthoracic echocardiography

CFR can be measured using Doppler transthoracic echocardiography (TTE) and correlates well with invasive Doppler and PET.w6 w14 The mid to distal left anterior descending artery (LAD) can be identified in a modified apical two-chamber view using a high frequency transducer and appropriate machine settings to identify low velocity flow. Pulse wave Doppler signals can be measured in the LAD at rest and during hyperaemia to calculate CFR.w14 This technique is feasible in most patients, including those who are obese, as it is less reliant on good acoustic windows due to the superficial location of the LAD.w6

### Myocardial contrast echo

Myocardial contrast echocardiography uses protein microbubbles that have a lower diameter than the red blood cell, resist arterial pressure and remain intravascular in the intact circulation. These qualities enable direct quantification of microvascular perfusion and allow absolute MBF as well as CFR to be calculated.^10^

## CMD in CKD: the evidence so far

A structured PubMed database search was carried out for articles between 1966 and 2019 using the keywords ‘coronary flow reserve’, ‘myocardial perfusion reserve’ or ‘coronary microvascular dysfunction’ combined with ‘chronic kidney disease’, ‘end-stage kidney disease’, ‘end-stage renal disease’ or ‘uraemic cardiomyopathy’. A total of 396 articles were identified. After removal of duplicates, 20 studies were considered relevant to this topic. Included studies are summarised in table 2.

**Table 2:** Summary of studies on coronary microvascular dysfunction in chronic kidney disease (CKD)

Study	Year	Country	Population	Modality	Findings
Ragosta *et al* 18	2004	USA	Controls (n=32) Patients with diabetes with no kidney disease (n=11) Patients with diabetic nephropathy (n=21)	Doppler angiography	Significantly lower CFR in patients with diabetic nephropathy compared with other two groups.
Tok *et al* 25	2005	Turkey	Controls (n=14) Patients on HD (n=10)	Doppler TTE	Significantly lower CFR in HD patients.
Chade *et al* 17	2006	USA	GFR >60 mL/min (n=481) GFR <60 mL/min (n=124)	Doppler angiography	Non-significant trend towards reduced CFR as eGFR falls.
Viganò *et al* 26	2007	Italy	Controls (n=17) Renal transplant recipients (n=25)	Doppler TTE	CFR impaired in half of cases.
Niizuma *et al* 24	2008	Japan	Controls (n=20) Patients on HD (n=21)	Doppler TTE	Significantly lower CFR in HD patients.
Caliskan *et al* 12	2008	Turkey	Controls (n=39) HD (n=48) Renal transplant recipients (n=27)	Doppler TTE	Significantly lower CFR in ESRD and in renal transplant recipients. Lower CFR in ESRD than renal transplant recipients.
Bezante *et al* 11	2009	Italy	Patients with hypertension and normal renal function (n=64) Patients with hypertension and renal impairment (n=12)	Doppler TTE	Significantly lower CFR in patients with hypertension and renal impairment.
Koivuviita *et al* 20	2009	Finland	Controls (n=10) CKD stages 3–5 (n=22)	PET	Non-significant trend towards reduced CFR as eGFR falls.
Turiel *et al* 27	2009	Italy	Controls (n=25) Renal transplant recipients (n=25)	Doppler TTE	Significantly lower CFR in renal transplant recipients compared with controls.
Bozbas *et al* 13	2009	Turkey	Controls (n=26) ESRD (n=30) Renal transplant recipients (n=30)	Doppler TTE	Significantly lower CFR in ESRD and in renal transplant recipients. Lower CFR in ESRD than renal transplant recipients.
Charytan *et al* 21	2010	USA	CKD stages 1–3 (n=435)	PET	Non-significant trend towards reduced CFR as eGFR falls
Akagun *et al* 28	2011	Turkey	Renal transplant recipients (n=20)	Doppler TTE	CFR <2 in 65%
Murthy *et al* 29	2012	USA	eGFR <60 mL/min (n=866)	PET	CFR <1.5 associated with increased risk of cardiac mortality.
Imamura *et al* 23	2014	Japan	Controls (n=15) CKD stages 1–5 (n=175)	Doppler TTE	Significant decrease in CFR as eGFR falls. Incremental reduction in CFR with albuminuria.
Shah *et al* 14	2016	USA	Dialysis-dependent patients (n=168)	PET	CFR <1.5 associated with increased risk of cardiac mortality.
Nakanishi *et al* 15	2013	Japan	eGFR <60 mL/min (n=139)	Doppler TTE	CFR <2 associated with worse cardiovascular outcomes.
Tona *et al* 30	2016	Italy	Simultaneous kidney pancreas transplant recipients (n=48)	Doppler TTE	Lower CFR associated with worse cardiovascular outcomes.
Paz *et al* 16	2017	USA	ESRD awaiting transplant (n=131)	PET	CFR <2 in 58.8% of patients with ESRD.
Charytan *et al* 22	2018	USA	Controls (n=198) CKD stages 1–5 (n=3748)	PET	Significant decrease in CFR as CKD stage increases.
Nelson *et al* 19	2019	USA	Controls (n=15) ESRD (n=15)	Doppler angiography	Significantly reduced CFR in ESRD compared with controls.

CFR, Coronary flow reserve; eGFR, estimated glomerular filtration rate; ESRD, end-stage renal disease; HD, haemodialysis; PET, positron emission tomography; TTE, transthoracic echocardiography.

There are limited conflicting data on coronary microvascular function in CKD. CMD appears common with prevalence rates of 24%–90%.^11–16^ The largest angiographic study examined CFR in 605 patients stratified as normal or reduced kidney function, using an arbitrary cut-off eGFR of 60 mL/min. Crude analysis indicated a reduced CFR in patients with CKD but this was not statistically significant after correction for age, gender and comorbidities including hypertension and diabetes.^17^ By contrast, smaller angiographic studies have shown a significant decrease in CFR in patients with diabetic nephropathy and in ESRD, compared with healthy controls.^18 19^

Using PET, a study of 10 controls and 22 patients with CKD stages 3–5 showed a trend towards reduced CFR with increasing stage of CKD, but this did not reach statistical significance.^20^ Similarly, retrospective calculation of CFR using PET in 435 patients with stages 1–3 CKD also found there was no significant difference in CFR after correction for cardiovascular risk factors such as age, sex and blood pressure.^21^ The largest PET study to date is a retrospective analysis of 3946 patients who underwent stress PET at a single US institution. This study demonstrated a significant decrease in CFR as renal function declined, with the largest drop being in patients with CKD stage 4 and no significant further drop in stage 5 or those on dialysis.^22^ In patients undergoing cardiovascular assessment for renal transplant by dipyridamole PET perfusion imaging, 59% of patients had a CFR <2 and even in those patients without any feature of infarction or ischaemia, 63% had abnormal CFR.^16^

Several studies have been performed using TTE. The relationship between albuminuria and CMD was investigated in a prospective Japanese study of 175 patients with CKD. Significant reductions in CFR with increasing stages of CKD were evident and patients with albuminuria, had significantly lower CFR at each stage of CKD.^23^ In patients with essential hypertension, the presence of CKD was associated with a 10-fold increase in the risk of CMD.^11^ Small studies of dialysis patients and controls have also shown reduced CFR measured by TTE in patients on haemodialysis compared with controls.^24 25^

There has been limited investigation into the effects of kidney transplantation on CFR. As kidney function is partially restored, one would expect an improvement in coronary microvascular function after kidney transplantation. This is suggested in cross-sectional data showing that CFR may be higher in transplant recipients compared with patients with ESRD.^12 13^ Possible explanations are that some of the microvascular changes seen in ESRD are reversible or alternatively that repeated haemodialysis causes microvascular dysfunction. Despite this, rates of CMD remain high after kidney transplant, with 8%–65% of renal transplant recipients having a CFR <2.^26–28^

The mechanisms of CMD in CKD are not fully understood. Patients with CKD demonstrate increased resting coronary flow but an impaired response to vasodilator stimuli, leading to reduced CFR.^11 14 18^ The reduced response to adenosine and other vasodilatory stimuli is likely to be due to factors such as reduced cross-sectional area of the microcirculation, increased coronary sympathetic arteriolar tone, endothelial dysfunction and a reduced smooth muscle response which may be due to defects at both receptor and post receptor levels.w9 w18 w19 Impaired myocardial vascular reserve and MBF autoregulation has been demonstrated in animal models of CKD.w20

## The prognostic role of reduced CFR in CKD

Several studies have investigated the relationship between reduced CFR and prognosis in CKD. These are mainly retrospective and included patients with comorbidities such as diabetes and hypertension that are known to affect CFR. In a retrospective analysis of 866 patients with moderate to severe CKD who underwent stress PET, even after adjustment for clinical risk factors, LV systolic function, extent of ischaemia and scar, a CFR below the median (<1.5) was associated with a 2.1-fold increased risk of cardiovascular mortality.^29^ The same investigators also retrospectively examined a cohort of 186 patients with dialysis dependent ESRD and again found that CFR below the median (1.4) was associated with a significant increased risk of all-cause and cardiovascular mortality over a median follow-up period of 3 years. Log transformed CFR was independently associated with all-cause and cardiovascular mortality.^14^ A prospective study using Doppler TTE assessed 139 patients with CKD, and identified that patients with CFR <2 had significantly higher rates of cardiac events and all-cause mortality even after adjustment for cardiovascular risk factors.^15^

There is very limited information on the role of CFR in predicting prognosis in renal transplant recipients. Data are conflicting with a small Turkish study (n=20) showing no prognostic role for CFR measurement in kidney transplant patients.^28^ By contrast, an Italian study of 48 patients who had undergone simultaneous kidney/pancreas transplant showed that a CFR <2 was associated with increased risk of major adverse cardiovascular events.^30^ However, it is difficult to draw any meaningful conclusions from these studies given the small numbers involved.

## Conclusions

CMD provides a plausible mechanism by which factors associated with impaired kidney function, including oxidative stress and inflammation, might result in myocardial damage and dysfunction leading to the syndrome of uraemic cardiomyopathy. Current data on CMD in uraemic cardiomyopathy are limited and conflicting, hampered by the retrospective design of most studies. Consequently, there is a need for well-designed prospective studies of CMD in CKD, to identify whether CMD might be a key mediator in the development of uraemic cardiomyopathy. Future studies will need to investigate the utility of strategies to prevent or reduce CMD and thus fibrosis and ventricular dysfunction in CKD. Possible agents that may be effective in in this regard might include new biological agents acting on inflammatory cytokines, antioxidants and, further down the pathway, specific antifibrotic drugs. Given the prevalence of CKD in the general population, a better understanding of the mechanisms behind uraemic cardiomyopathy is a vital step towards improving the significant cardiovascular morbidity and mortality seen in CKD.
