# Supplementary material for: Coronary microvascular dysfunction: a key step in the development of uraemic cardiomyopathy?
Source: Heart. 2019 Jun 25;105(17):1302–9. doi: 10.1136/heartjnl-2019-315138 (PMC6711343; doi:10.1136/heartjnl-2019-315138)
Supplement: Supplementary data [file heartjnl-2019-315138supp001.docx]

**Supplementary reference list**

S1. Coresh J, Selvin E, Stevens LA, *et al.* Prevalence of chronic kidney disease in the United States. *JAMA* 2007;**298**:2038–47.http://dx.doi.org/10.1001/jama.298.17.2038

S2. Radenkovic D, Weingärtner S, Ricketts L, *et al.* T1 mapping in cardiac MRI. *Hear Fail Rev* 2017;**22**:415–30. doi:10.1007/s10741-017-9627-2

S3. Diao K, Yang Z, Xu H, *et al.* Histologic validation of myocardial fibrosis measured by T1 mapping : a systematic review and meta-analysis. *J Cardiovasc Magn Reson* 2016;**18**:1–11. doi:10.1186/s12968-016-0313-7

S4. Edwards NC, Hirth A, Ferro CJ, *et al.* Subclinical Abnormalities of Left Ventricular Myocardial Deformation in Early-Stage Chronic Kidney Disease: The Precursor of Uremic Cardiomyopathy? *J Am Soc Echocardiogr* 2008;**21**:1293–8. doi:https://doi.org/10.1016/j.echo.2008.09.013

S5. Moreo A, Ambrosio G, Chiara B De, *et al.* Influence of Myocardial Fibrosis on Left Ventricular Noninvasive Assessment by Cardiac Magnetic Resonance and Echo. *Circ Cardiovasc Imaging* 2009;**2**:437–43. doi:10.1161/CIRCIMAGING.108.838367

S6. Shah SJ, Lam CSP, Svedlund S, *et al.* Prevalence and correlates of coronary microvascular dysfunction in heart failure with preserved ejection fraction: PROMIS-HFpEF. *Eur Heart J* 2018;**39**:3439–50. doi:10.1093/eurheartj/ehy531

S7. Chilian WM. Coronary Microcirculation in Health and Disease Summary of an NHLBI Workshop. *Circulation* 1997;**95**:522–8. doi:http://www.ncbi.nlm.nih.gov/pmc/articles/PMC4037233/

S8. Muller-delp JM. The Coronary Microcirculation in Health and Disease. *ISRN Physiol* 2013;**2013**. doi:https://doi.org/10.1155/2013/238979

S9. Amann K, Breitbach M, Ritz E, *et al.* Myocyte/capillary mismatch in the heart of uremic patients. *J Am Soc Nephrol* 1998;**9**:1018–22.http://eutils.ncbi.nlm.nih.gov/entrez/eutils/elink.fcgi?dbfrom=pubmed&id=9621284&retmode=ref&cmd=prlinks%5Cnpapers3://publication/uuid/609789A0-169D-4D2F-97C6-95CA5C75B60D

S10. Amann K, Wiest G, Zimmer G, *et al.* Reduced capillary density in the myocardium of uremic rats--a stereological study. *Kidney Int* 1992;**42**:1079–85. doi:10.1038/ki.1992.390

S11. Cecchi F, Olivotto I, Gistri R, *et al.* Coronary Microvascular Dysfunction and Prognosis in Hypertrophic Cardiomyopathy. *N Engl J Med* 2003;**349**:1027–35.http://www.nejm.org/doi/abs/10.1056/NEJMoa025050%5Cnpapers3://publication/doi/10.1056/NEJMoa025050

S12. Nelson MD, Wei J, Bairey Merz CN. Coronary microvascular dysfunction and heart failure with preserved ejection fraction as female-pattern cardiovascular disease: The chicken or the egg? *Eur Heart J* 2018;**39**:850–2. doi:10.1093/eurheartj/ehx818

S13. Layland J, Carrick D, Lee M, *et al.* Adenosine: Physiology, pharmacology, and clinical applications. *JACC Cardiovasc Interv* 2014;**7**:581–91. doi:10.1016/j.jcin.2014.02.009

S14. Caiati C, Montaldo C, Zedda N, *et al.* Validation of a new noninvasive method (contrast-enhanced transthoracic second harmonic echo Doppler) for the evaluation of coronary flow reserve: Comparison with intracoronary Doppler flow wire. *J Am Coll Cardiol* 1999;**34**:1193–200. doi:10.1016/S0735-1097(99)00342-3

S15. Pijls NH, De Bruyne B, Smith L, *et al.* Coronary Thermodilution to Assess Flow Reserve. Validation in Humans. *Circulation* 2002;**105**:2482–6. doi:10.1161/01.CIR.0000017199.09457.3D

S16. Kato S, Saito N, Nakachi T, *et al.* Stress Perfusion Coronary Flow Reserve Versus Cardiac Magnetic Resonance for Known or Suspected CAD. *J Am Coll Cardiol* 2017;**70**:869–79. doi:10.1016/j.jacc.2017.06.028

S17. Liu A, Wijesurendra RS, Francis JM, *et al.* Adenosine Stress and Rest T1 Mapping Can Differentiate between Ischemic, Infarcted, Remote, and Normal Myocardium Without the Need for Gadolinium Contrast Agents. *JACC Cardiovasc Imaging* 2016;**9**:27–36. doi:10.1016/j.jcmg.2015.08.018

S18. Kozàkovà M, Palombo C, Pratali L, *et al.* Mechanisms of Coronary Flow Reserve Impairment in Human Hypertension. *Hypertension* 1997;**29**:551–9. doi:10.1161/01.HYP.29.2.551

S19. Morris STW, McMurray JJV, Spiers A, *et al.* Impaired endothelial function in isolated human uremic resistance arteries. *Kidney Int* 2001;**60**:1077–82. doi:10.1046/j.1523-1755.2001.0600031077.x

S20. Kingma Jr. JG, Simard D, Voisine P, *et al.* Impact of Chronic Kidney Disease on Myocardial Blood Flow Regulation in Dogs. *Nephron Exp Nephrol* 2014;**126**:175–82. doi:10.1159/000362090
